# SEOM clinical guidelines for the treatment of head and neck cancer (2017)

**DOI:** 10.1007/s12094-017-1776-1

**Published:** 2017-11-20

**Authors:** L. C. Iglesias Docampo, V. Arrazubi Arrula, N. Baste Rotllan, A. Carral Maseda, B. Cirauqui Cirauqui, Y. Escobar, J. J. Lambea Sorrosal, M. Pastor Borgoñón, A. Rueda, J. J. Cruz Hernández

**Affiliations:** 1 Hospital Universitario 12 de Octubre, Madrid, Spain; 2 Hospital de Basurto, Bilbao, Spain; 3 Hospital Universitario Vall d´Hebrón, Barcelona, Spain; 4 Hospital Universitario Lucus Augusti, Lugo, Spain; 5 Hospital Universitari Germans Trias i Pujol, Badalona, Spain; 6 Hospital General Universitario Gregorio Marañón, Madrid, Spain; 7 Hospital Clínico Universitario Lozano Blesa, Zaragoza, Spain; 8 Hospital Universitari I Politècnic la Fe, Valencia, Spain; 9 Complejo Hospital Costa del Sol, Málaga, Marbella, Spain; 10 Hospital Universitario de Salamanca, Salamanca, Spain

**Keywords:** Head and neck cancer, Guidelines, HPV, Induction chemotherapy

## Abstract

Head and neck cancer (HNC) is defined as malignant tumours located in the upper aerodigestive tract and represents 5% of oncologic cases in adults in Spain. More than 90% of these tumours have squamous histology. In an effort to incorporate evidence obtained since 2013 publication, Spanish Society of Medical Oncology (SEOM) presents an update of HNC diagnosis and treatment guideline. The eighth edition of TNM classification, published in January 2017, introduces important changes for p16-positive oropharyngeal tumours, for lip and oral cavity cancer and for N3 category. In addition, there are new data about induction chemotherapy and the role of immunotherapy in HNC.

## Introduction

Head and neck cancer (HNC) is defined as malignant tumours located in the upper aerodigestive tract (paranasal sinuses, nasopharynx, oropharynx, hypopharynx, larynx, oral cavity, nostrils and salivary glands).

It is necessary to emphasize that the most important risk factor continues to be tobacco along with alcohol, but the infection by human papillomavirus is key in the origin of some of these tumours and confers them special characteristics that possibly in the future condition its treatment.

It is a neoplasm with a high possibility of cure if it is diagnosed in early stages, but unfortunately two-thirds of the patients are diagnosed at an advanced locoregional stage (stages III and IV, without metastasis). More than 90% of these tumours have a squamous histology. In this guide we only talk about them.

In Spain, HNC represents 5% of all new cancer diagnoses in adults, being the sixth frequency neoplasia (fifth in men), with an incidence similar to the European median, and a mortality rate of three points below the European median [1].

A multidisciplinary team, bringing together all professionals who specialize in the diagnosis and treatment of these tumours, will make the decision to establish the best sequence of individualized treatment for each patient.

Within what is known as HNC, each location has a clinical presentation, staging, prognosis and different therapeutic approach. As this is a general guide, the particularities of each subsite will not be dealt with.

## Methodology

Methodology SEOM guidelines have been developed with the consensus of ten oncologists from the Spanish Group for the Treatment of Head and Neck Tumors (TTCC) and SEOM. To assign a level and quality of evidence and a grade of recommendation to the different statements of this treatment guideline, the Infectious Diseases Society of America-US Public Health Service Grading System for Ranking Recommendations in Clinical Guidelines was used (Table 1). The final text has been reviewed and approved by all authors.

**Table 1:** Strength of recommendation and quality of evidence score

Category, grade	Definition
Strength of recommendation	
A	Good evidence to support a recommendation for use
B	Moderate evidence to support a recommendation for use
C	Poor evidence to support a recommendation
D	Moderate evidence to support a recommendation against use
E	Good evidence to support a recommendation against use
Quality of evidence	
I	Evidence from ≥ 1 properly randomized, controlled trial
II	Evidence from ≥ 1 well-designed clinical trial, without randomization; from cohort or case controlled analytic studies (preferably from > 1 centre); from multiple time series; or from dramatic results from uncontrolled experiments
III	Evidence from opinions of respected authorities, based on clinical experience, descriptive studies, or reports of expert committees

## Diagnosis and staging

Recording a good clinical history and following a methodology for the diagnosis of HNC should be inherent in all good clinical practice. We describe the essential points of any clinical history of a patient with HNC, not forgetting that a patient may have other symptoms or diseases.

Accurate staging is crucial for determining the appropriate approach and for tailoring therapy to each individual patient. In HNC, the following staging process is recommended:

- Complete history and physical examination.
- Complete examination of the head and neck area (endoscopic examination).
- Histological diagnosis:
  Primary tumour biopsy.
  Lymph node puncture for cytological specimen.
  Human papilloma virus determination in oropharynx and oral cavity tumours.
- Imaging diagnosis:
  Cervical computed tomography (CT) or magnetic resonance (MR).
  Chest imaging (X-ray) or computed tomography (CT) preferably.
  Esophageal–gastric contrast study or esophagoscopy in case of dysphagia.
  Consider positron emission tomography (PET) for stage III–IV disease (patients with definitive treatment intention and high risk of metastases).
- Functionalism evaluation: swallowing, phonation, breathing, odontology and nutritional status.
- Special evaluations if needed: psychological and social situation, prevention and cessation of cigarette smoking or alcohol dependence, etc.

The TNM classification is the internationally accepted system for tumour staging. Stage at diagnosis predicts survival rates and guide management. The eighth edition of TNM classification was published in January, 2017 [2, 3]; however, its implementation is scheduled for January, 2018. The aim of this article is to be used for the managing of head and neck tumours in the next years. Therefore, the eighth classification is detailed (Tables 2 and 3).

**Table 2:** T category for the different locations

A. Lip and oral cavity	
T1	Tumour 2 cm or less in greatest dimension and 5 mm or less depth of invasion
T2	Tumour 2 cm or less in greatest dimension and more than 5 mm but not more than 10 mm depth invasion or Tumour more than 2 cm but not more than 4 cm in greatest dimension and depth if invasion no more than 10 mm
T3	Tumour more than 4 cm in greatest dimension or more than 10 mm in depth invasion
T4a (lip)	Tumour invades through cortical bone, inferior alveolar nerve, floor of mouth, or skin
T4a (oral cavity)	Tumour invades through the cortical bone of the mandible or maxillary sinus, or invades the skin of the face
T4b	Tumour invades masticator space, pterygoid plates, or skull base, or encases internal carotid artery

B. Oropharynx p16*-*negative^a^	
T1	Tumour 2 cm or less in greatest dimension
T2	Tumour more than 2 cm but not more than 4 cm in greatest dimension
T3	Tumour more than 4 cm in greatest dimension extension to lingual surface of epiglottis
T4a	Tumour invades any of the following: larynx, deep/extrinsic muscle or tongue, medial pterygoid, hard palate, or mandible
T4b	Tumour invades any of the following: lateral pterygoid muscle, pterygoid plates, lateral nasopharynx, skull base; or encase carotid artery

C. Hypopharynx	
T1	Tumour limited to one subsite of hypopharynx and /or 2 cm or less in greatest dimension
T2	Tumour invades more than one subsite of hypopharynx or an adjacent site, or measures more than 2 cm but not more than 4 cm in greatest dimension, without fixation of hemilarynx
T3	Tumour more than 4 cm in greatest dimension or with fixation of hemilarynx or extension to oesophagus
T4a	Tumour invades any of the following: thyroid/cricoid cartilage, hyoid bone, thyroid gland, oesophagus, central compartmental soft tissue
T4b	Tumour invades prevertebral fascia, encases carotid artery, or invades mediastinal structures

D. Larynx: supraglottis	
T1	Tumour limited to one subsite of supraglottis with normal vocal cord mobility
T2	Tumour invades mucosa of more than one adjacent subsite of supraglottis or glottis or region outside of supraglottis without fixation of the larynx
T3	Tumour limited to larynx with vocal cord fixation and/or invades any of the following: postcricoid area, pre-epiglottic space, paraglottic space, and/or inner cortex of thyroid cartilage
T4a	Tumour invades through the thyroid cartilage and/or invades tissues beyond the larynx
T4b	Tumour invades prevertebral space, encases carotid artery, or mediastinal structures

E. Larynx: glottis	
Tl	Tumour limited to vocal cords with normal mobility
	T1a Tumour limited to one vocal cord
	T1b Tumour involves both vocal cords
T2	Tumour extends to supragottis and/or subglottis, and/or with impairs vocal cord mobility
T3	Tumour limited to larynx with vocal cord fixation and/or invades paraglottic space, and/or inner cortex of the thyroid cartilage
T4a	Tumour invades through the outer cortex of thyroid cartilage and/or invades tissues beyond the larynx
T4b	Tumour invades prevertebral space, encases carotid artery, or mediastinal structures

F. Larynx: subglottis	
Tl	Tumour limited to subglottis
T2	Tumour extends to vocal cord(s) with normal or impaired mobility
T3	Tumour limited to larynx with vocal cord fixation
T4a	Tumour invades cricoid or thyroid cartilage, and/or invades tissues beyond the larynx
T4b	Tumour invades prevertebral space encases carotid artery, or mediastinal structures

^a^ ln oropharynx p16-positive tumours T4a and T4b categories are classified as T4

**Table 3:** N category for all locations

A. Regional lymph nodes (except oropharynx pl6-positive)	
NX	Regional lymph nodes cannot be assessed
NO	No regional lymph node metastasis
N1	Metastasis in a single ipsilateral lymph node, 3 cm or less in greatest dimension without extranodal extension
N2a	Metastasis in a single ipsilateral lymph node more than 3 cm but no more than 5 cm in greatest dimension without extranodal extension
N2b	Metastasis in multiple ipsilateral lymph nodes, none more than 6 cm in greatest dimension without extranodal extension
N2c	Metastasis in bilateral or contralateral lymph nodes, none more than 6 cm in greatest dimension without extranodal extension
N3a	Metastasis in a lymph node more than 6 cm in greatest dimension without extranodal extension
M3b	Metastasis in a single or multiple lymph nodes with clinical extranodal extension

B. Regional lymph nodes in oropharynx p16-positive tumours	
nX	Regional lymph nodes cannot be assessed
N0	No regional lymph node metastasis
N1	Unilateral metastasis, in lymph node(s), all 6 cm or less in greatest dimension
N2	Contralateral or bilateral metastasis in lymph node(s), all 6 cm or less in greatest dimension
N3	Metastasis in lymph node(s) greater than 5 cm in dimension

The main change to the seventh edition is the separate classification for p16-positive oropharyngeal tumours. In the T category, T4a and T4b are pooled as T4 in p16-positive oropharyngeal tumours (Table 2 B). In addition, N category has been reclassified (Table 3 B).

Other modifications in the eight edition are as follows: T category (T1–T3) of lip and oral cavity includes the extent of depth invasion (Table 2 A) and N3 category for all locations has been subdivided into N3a and N3b according to extranodal extension (in N1 and N2 categories lack of extranodal extension is required; Table  3 A).

The overall stage of the tumour is complete with the definition of the presence (M0) or absence (M1) of distant metastasis. The AJCC stage groupings are the result of combining T, N and M categories.

## Early disease (clinical stage I–II) treatment

Both surgery and radiotherapy (RT) (external or brachytherapy) provide similar locoregional control and survival outcomes, but they have not been compared in randomized trials [4, 5]. The choice of treatment modality depends on the functional outcome, the patient’s wishes, the possibility of an adequate follow-up, the patient’s general condition, and the likelihood of developing a second primary tumour (e.g., younger smoking patients use the surgical option not to jeopardize further treatment).

Curative surgery is the preferred option for cancer of the oral cavity and involves resection of tumour with an appropriate safety margin and subsequent reconstruction [II, B]. Elective neck dissection offers improved overall and disease-free survival compared with therapeutic neck dissection [I, A] [6]. Sentinel node lymph node biopsy may be indicated for small cancers to avoid morbidity [II, B] [7].

Oropharyngeal carcinoma should ideally be treated with single-modality therapy, either primary surgery or RT. Radical RT is a good option (a total dose equivalent of 70 Gy in 35 fractions is used) [II, B]. Prophylactic RT should be given to the ipsilateral cervical lymph nodes for lateralized tumours and to both sides of the neck for non-lateralised tumours [II, B]. Surgery should usually be carried out transorally, either by transoral laser microsurgery (TLM) or transoral robotic surgery (TORS). Oncologic results after transoral resection of the oropharynx appear to be comparable to open surgery [II, B] [8], which is associated with increased morbidity and treatment complications. Patients having surgery to the primary should also undergo ipsilateral selective neck dissection. Dissection of the contralateral neck may also be considered in tumours arising at or very near the midline [II, B].

Radiotherapy or TLM are the two most commonly used treatment modalities in early laryngeal cancer [II, B]. Individual treatment selection depends on patient and tumour factors and local expertise. Single-modality treatment is sufficient and combining surgery with RT should be avoided as functional outcomes (and perhaps survival in the context of incompletely resected tumour) may be compromised by combined modality therapy. Open surgical procedures are used less commonly today; however, they provide an option for the treatment of tumours which are not accessible to TLM. Elective treatment of the neck is not recommended because of the very low risk of occult nodal disease [III, C].

Early lesions of the hypopharynx can be treated with equal effectiveness with surgery or radiation [9]. Occult nodal disease is present in 30–40% of patients, so any treatment plan should include elective treatment of the cervical nodes [II, B].

## Locally advanced disease (clinical stages III, IV-A, IV-B) treatment

In all cases there must be a multidisciplinary assessment to decide the best treatment option for each patient.

This type of tumours is divided into two groups: resectable and unresectable. There is no universally accepted definition of unresectability but some anatomical criteria are considered unequivocal (involvement of skull base, cervical vertebrae, prevertebral muscles, brachial plexus, mediastinal spread, involvement of the nasopharynx, fixed tumour to collarbone). Final decision depends on institution and surgeon abilities. Furthermore, if surgical team foresees the impossibility of achieving complete excision with adequate margins and/or functional and/or aesthetic sequelae of surgery are not acceptable and/or little expectation of surgical cure and/or high-risk surgery due to age or comorbidities, patient should be considered not able to be suitably operated.

The patient’s nutritional status must be corrected and maintained. Dental rehabilitation is indicated before radiotherapy. Treatment depends on primary tumour location and extension.

A. Resectable locally advanced disease (III–IV-A) (Fig. 1)

**Fig. 1:**
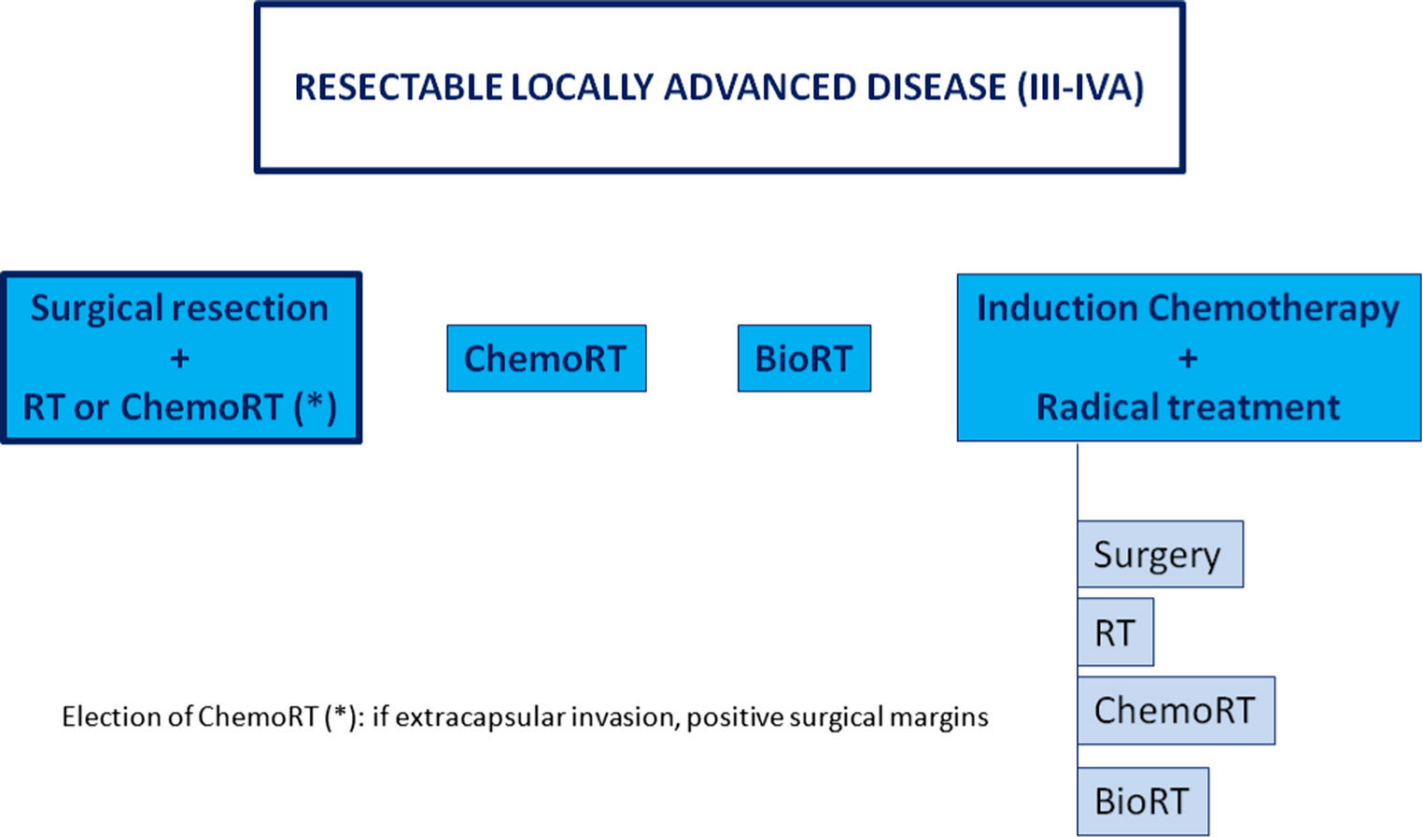
Treatment algorithm for resectable locally advanced disease (III–IVA)
1. Surgical resection followed by radiotherapy (IA) or chemoradiotherapy (IA). Adjuvant concurrent chemoradiotherapy (with three-weekly administration cisplatin 100 mg/m^2^ days 1, 22, 43) is recommended in patients with high-risk pathological features: extracapsular lymph node extension and/or affected margins (IA) [10, 11].
2. Chemoradiation treatment is preferred for patients that are not candidates for or refuse conservative surgery. The standard schedule is cisplatin (100 mg/m^2^ days 1, 22, 43) [12] (IA). Bioradiotherapy with cetuximab is an alternative treatment (400 mg/m^2^ at initial dose day −8 followed by 250 mg/m^2^ weekly concurrent) for patients with some contraindication for cisplatin such as neuropathy, nephropathy, heart disease and hearing loss [13] (IA).
3. Induction chemotherapy (ICT) can be used, with TPF schedule (three-weekly administration Cisplatin 75 mg/m^2^ + Docetaxel 75 mg/m^2^ + 5-FU 750 mg/m^2^/d continue infusion 96 h). However, an improvement in overall survival with the incorporation of ICT compared to chemoradiotherapy with cisplatin has not been established. Nowadays, there is not any standard locoregional treatment (radiotherapy, chemoradiotherapy, bioradiotherapy) established in responder patients to ICT and should be performed according to the response and tolerance to ICT [14].

Evaluation of response after ICT:

- Complete response: disappearance of all clinically tumour burden.
- Partial response: ≥ 50% reduction of primary tumour without lymph node progression.
- Stable disease: neither sufficient shrinkage to qualify for PR nor sufficient increase to qualify for progression disease.
- Progression disease: increase of tumour burden.

After locoregional treatment: salvage neck dissection should be considered in patients with residual lymph node disease and complete response of primary tumour.

**Specific recommendations on locally advanced disease by anatomic site:**

**Fig. 2:**
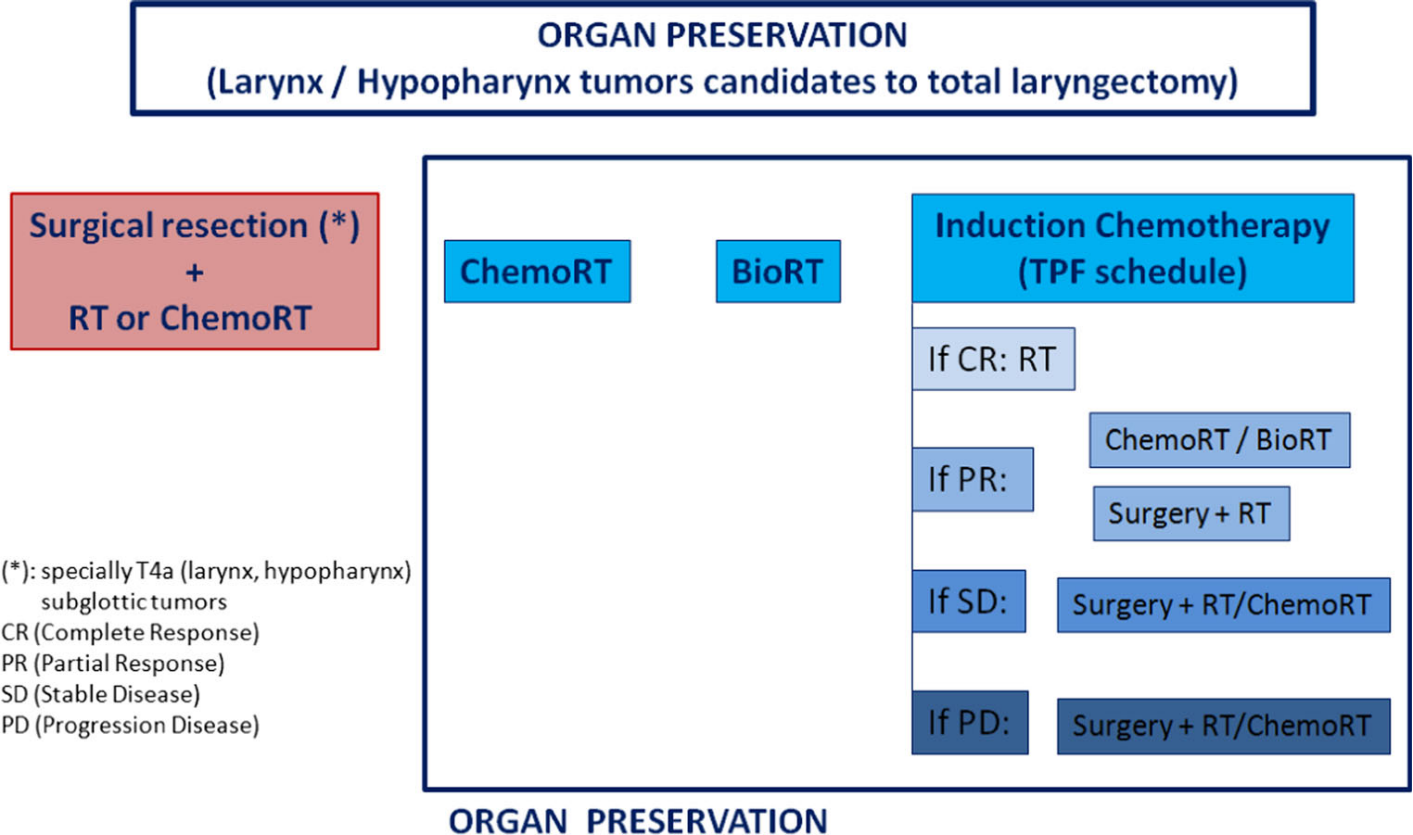
Larynx preservation algorithm (resectable locally advanced disease)

1. Hypopharynx: three options could be considered:

- Surgical resection (total pharyngo-laryngectomy + neck dissection) followed by radiotherapy (IA) or chemoradiotherapy (IA) if high-risk recurrence of pathological factors. Specially T4a.
- Concurrent chemoradiotherapy with three-weekly cisplatin is recommended if patient refuses surgery (IA). If cisplatin cannot be administered: cetuximab concurrent to radiotherapy (IA).
- Induction chemotherapy with TPF schedule:
  - If complete response → radiotherapy (based on initial stage) ± cisplatin/cetuximab (based on ICT toxicity).
  - If partial response → surgery followed by radiotherapy or chemoradiotherapy. If the main objective is organ preservation, consider concomitant RT (with cisplatin or cetuximab) (IIB).
  - If stable disease or progression → surgery (including neck dissection) followed by radiotherapy or chemoradiotherapy.

2. Larynx: three options could be considered (Fig. 2):

- Surgical resection (total versus partial laryngectomy + neck dissection) followed by radiotherapy (IA) or chemoradiotherapy (IA) if high-risk recurrence of pathological factors.
  - Specially T4a.
  - For the most part of subglottic tumours.
- Concurrent chemoradiotherapy with three-weekly cisplatin is recommended if patient refuses surgery (IA). If cisplatin cannot be administered: cetuximab concurrent to radiotherapy (IA).
- Induction chemotherapy with TPF schedule (except for subglottic tumours) [15]:
  - If complete response → radiotherapy.
  - If partial response → concomitant RT (with cisplatin or cetuximab) (IIB) or consider surgery followed by radiotherapy.
  - If stable disease or progression → surgery (including neck dissection) followed by radiotherapy or chemoradiotherapy.

3. Oropharynx:

- Concurrent chemoradiotherapy with three-weekly cisplatin is recommended (IA). If cisplatin cannot be administered: cetuximab concurrent to radiotherapy (IA).
- Consider induction chemotherapy with TPF schedule, only in those patients N bulky and fast tumour growth, individualizing benefit and toxicity.

**Fig. 3:**
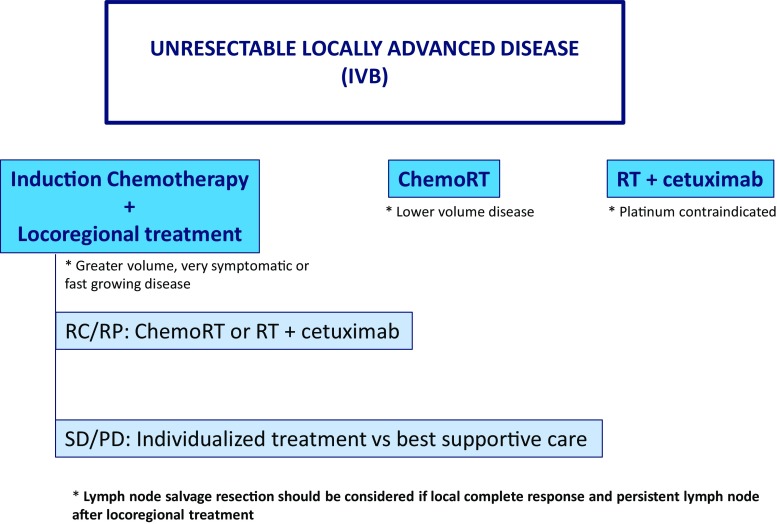
Treatment algorithm for unresectable locally advanced disease (IV-B)

B. Unresectable locally advanced disease (IV-B) (Fig. 3)

Different therapeutic strategies have been explored in this scenario

1. Concomitant chemoradiotherapy with three-weekly cisplatin. Several studies have demonstrated benefit in locoregional control and overall survival over radiotherapy alone with a significant increase in acute and chronic toxicity.
2. Concomitant radiotherapy and cetuximab have shown a benefit in locoregional control and overall survival compared to radiotherapy alone with a better toxicity profile compared to chemotherapy. It should be considered if the use of cisplatin is contraindicated such as neuropathy, nephropathy, heart disease and hearing loss.
3. Induction chemotherapy followed by locoregional treatment. This option has been reconsidered, especially in patients who require rapid response or are at increased risk of distant metastases.

**Recommendations for unresectable locally advanced disease (IV-B)**

1. Induction chemotherapy followed by locoregional treatment

TPF × 3 cycles (IA) if ECOG 0–1 and good renal and liver function.

This strategy is recommended in greater volume (N3, N2c, important N2b, T4b), very symptomatic and fast-growing locally advanced disease.

After induction chemotherapy:

- If CR/PR: RT + cisplatin (IIB) or RT + cetuximab (IIB). TTCC group performed a trial which prelimimary results are inconclusive (because the required number of events have not yet been observed) but both arms show a good locoregional control of 50% at 3 years [16]. The treatment’s choice will be based on toxicities during induction chemotherapy and on prediction of tolerance to platinum-based sequential chemoradiotherapy.
- If SD or PD: individualized treatment or best supportive care (includes palliative radiotherapy).

2. Concomitant chemoradiotherapy with cisplatin 100 mg/m^2^ days 1, 22, 43 (IA).
  Recommended in lower volume locally advanced disease (T4a, T3, N1-2a).
3. Concomitant bioradiotherapy and cetuximab (IA) (400 mg/m^2^ at initial dose day −8 followed by 250 mg/m^2^ weekly concurrent) for patient not eligible for platinum chemoradiotherapy.

In case of local complete response and persistent lymph node after locoregional treatment, lymph node salvage resection should be considered (IVD).

## Recurrent and metastatic disease treatment

The multidisciplinary team will assess the possibility of salvage surgery (operable tumour) or re-irradiation with or without chemotherapy/cetuximab. In the presence of oligometastatic disease, treatment with curative intent should also be discussed.

Once this option is discarded the treatment of choice is palliative chemotherapy:

### First-line treatment

1. Chemotherapy-naïve patients
  - In the patient with a performance status of 0/1 the first choice is the combination of cisplatin, 5-fluorouracil, and cetuximab (EXTREME protocol) [17]. If the patient is medically unfit to receive cisplatin the use of carboplatin may be an option. Cetuximab should be maintained until progression or unacceptable toxicity.
  - If the patient cannot be treated with platinum (concomitant disease, previous treatment, etc.) or patients with PS 2, the treatment of choice is best supportive treatment of symptoms. In these patients, the combination ERBITAX (paclitaxel plus cetuximab) should be considered [18].
  - The treatment of choice for patients with PS 3/4 is best supportive care of symptoms.
2. Patients who have received chemotherapy for locoregional disease
  - Patients with progressive disease more than 6 months after locoregional treatment can be treated like chemotherapy-naïve patients.
  - Patients with progressive disease within 6 months after last cisplatin dose should not receive cisplatin or carboplatin. ERBITAX combination or second-line therapy should be considered.

### Second-line treatment

Inmunotherapy with nivolumab [19] (Level of evidence I, A or pembrolizumab [20, 21] (Level II, B) has become the standard of care. PD-L1 positive tumours seem to benefit the most.

If it is not possible to use immunotherapy, considering using agents such as taxanes, methotrexate, cetuximab or gemcitabine. If bad PS, only support treatment should be considered.

All patients should be recommended including in clinical trials if available.
